# Current cell models for bioengineering a salivary gland: a mini-review of emerging technologies

**DOI:** 10.1111/j.1601-0825.2012.01958.x

**Published:** 2013-04

**Authors:** J Nelson, K Manzella, OJ Baker

**Affiliations:** Department of Oral Biology, School of Dental Medicine, University at Buffalo, The State University of New YorkBuffalo, NY, USA

**Keywords:** cell line, progenitor cells, primary culture, salivary gland dysfunction

## Abstract

Saliva plays a major role in maintaining oral health. Patients afflicted with a decrease in saliva secretion (symptomatically, xerostomia) exhibit difficulty in chewing and swallowing foods, tooth decay, periodontal disease, and microbial infections. Despite recent improvements in treating xerostomia (e.g., saliva stimulants, saliva substitutes, and gene therapy), there is a need of more scientific advancements that can be clinically applied toward restoration of compromised salivary gland function. Here we provide a summary of the current salivary cell models that have been used to advance restorative treatments via development of an artificial salivary gland. These models represent initial steps toward clinical and translational research, to facilitate creation of clinically safe salivary glands. Further studies in salivary cell lines and primary cells are necessary to improve survival rates, cell differentiation, and secretory function. Additionally, the characterization of salivary progenitor and stem cell markers are necessary. Although these models are not fully characterized, their improvement may lead to the construction of an artificial salivary gland that is in high demand for improving the quality of life of many patients suffering from salivary secretory dysfunction.

## Introduction

Hyposalivation is a significant clinical concern, as decreased saliva production leads to dental caries, periodontitis, microbial infections, and difficulties with basic oral functions (*e.g*., speaking, mastication, and swallowing), all of which significantly reduce the quality of life for afflicted patients (Thomson *et al*, 2006; Lawrence *et al*, 2008). Major causes of hyposalivation include (i) Sjögren’s Syndrome (SS), an autoimmune disease affecting approximately 1% of the population (Pillemer *et al*, 2001); (ii) γ-irradiation therapy administered to approximately 5% of the patients with head and neck cancer diagnosed each year in the United States (Jensen *et al*, 2010); (iii) side effects of medications used by a large portion of the population; and (iv) ectodermal dysplasias, a group of developmental disorders mainly affecting ectodermal tissues and organs (Pinheiro and Freire-Maia, 1994; Nordgarden *et al*, 2003; Clauss *et al*, 2008). Current treatments for hyposalivation are limited to (i) patient education, diet, and lifestyle modifications; (ii) prevention of dental and oral mucosal diseases; (iii) management of symptoms; (iv) sialogogues or salivary gland stimulants (e.g., the muscarinic receptor agonists pilocarpine and cevimeline) that induce saliva secretion from residual acinar cells (Vissink *et al*, 2010); and (v) use of artificial saliva (Silvestre *et al*, 2009). However, given that these therapies target surface-level symptoms and provide only temporary relief, development of an alternative treatment to provide a more permanent effect is essential. An *in vivo* gene therapy strategy involving viral vector-mediated transfer of the aquaporin-1 cDNA to irradiation-damaged salivary glands has been successfully tested in two pre-clinical models (irradiated rats and miniature pigs), as well as demonstrated its safety in a large toxicology and biodistribution study (Baum *et al*, 2009). This study represents an important advancement to treat secretory dysfunction; however, improvement of this therapeutic approach is required as incorporation of vital constituents of normal saliva (i.e., α-amylase and mucin) will be necessary in the future.

The development of a viable artificial salivary gland is a novel option in bringing relief to many patients afflicted with hyposalivation. Ideally, autologous primary cells should be used clinically (Hoekstra and Chamuleau, 2002). Hypothetically, a patient’s healthy tissue could be extracted prior to radiation therapy. During treatment, the cells could be grown on a scaffold and implanted back into the patient (Redman, 2008). In cases of severely damaged or absent salivary glands, however, this is not an option. These individuals would need to rely on an artificial salivary gland grown from donor cells. However, cells in primary cultures display slow growth, de-differentiation, and a finite lifespan (Redman *et al*, 1988; Yeh *et al*, 1991; Quissell *et al*, 1994a, b). Consequently, growing attention has been given to the use of salivary cell lines in developing an artificial gland (Demeter *et al*, 2009; Aure *et al*, 2010; Maria *et al*, 2011a, b). Currently, no cell line fully recapitulates the morphological and functional features of the native salivary acinar cells (Warner *et al*, 2008). Furthermore, cell lines are potentially tumorogenic. However, a study showed that the human submandibular gland (HSG) cell line could be transfected with a herpes simplex virus thymidine kinase (HSV-tk) suicide gene to provide additional safety for use in an artificial salivary gland. Unfortunately, the cell survival rate using this technology was low and needs to be optimized (Aframian *et al*, 2001). Therefore, we believe that salivary gland cell lines are good models for understanding physiology, behavior, pathological processes, genetic manipulations, and proof of concepts (See Table 1). However, with the current technology, available salivary cell lines are not ready to be used for implantation *in vivo*. In this review, we evaluate the potential advantages and limitations of commonly used salivary cell models.

**Table 1:** Cell models utilized in the development of an artificial salivary gland. This table summarizes the current cell models used in the design of cell constructs that may provide insight for engineering an artificial salivary gland

Cell line	Source	Immortalization action	Ability to acihieve polarity	Amylase expression	Ability to secrete fluid	References
HSY	Huma parotid adenocarcinoma	Tumor derived	+	+	+	Yanagawa *et al* (1986); Hayashi *et al* (1987)
HSG	Irradiated human submandibular gland intercalated duct cells	Tumor derived	+	+	+	Shirasuna *et al* (1981)
SMIE	Rat submandibular gland	12S E1A adenovirus gene product	+	−	+	He *et al* (1990, 1998))
RSMT-A5	Rat submandibular gland	3-Methylcholanthrene	−	−	−	Brown *et al* (1973); He *et al* (1989)
SMG-C6	Rat submandibular gland	Transformed with a replication-deficient Simian Virus (pSV40) construct	+	a	a	Quissell *et al* (1997)
SMG-C10	Rat submandibular gland	Transformed with a replication-deficient Simian Virus (pSV40) construct	+	a	a	Quissell *et al* (1997)
Par-C10	Rat parotid gland	Transformed with a replication-deficient Simian Virus (pSV40) construct	+	−	+	Quissell *et al* (1997, 1998); Baker *et al* (2010)
Par-C5	Rat parotid gland	Transformed with a replication-deficient Simian Virus (pSV40) construct	+	−	−	Quissell *et al* (1998)
Primary cells progenitor cells	Human submandibular and parotid glands	None	+	+	+	Pradhan *et al* (2010); Feng *et al* (2009)

a Not yet determined.

## Tumor-derived cell lines

### HSY

HSY is a neoplastic epithelial cell line that was established from athymic mice tumors after transplantation with surgical specimens of a human parotid gland adenocarcinoma (Yanagawa *et al*, 1986; Hayashi *et al*, 1987). Structurally, HSY cells are cuboidal in shape and show papillary infoldings by cytoplasmic processes and microvilli on their free border (Nagamine *et al*, 1990; Hayashi *et al*, 1987). These cells are able to establish intercellular connections such as desmosomes and tight junctions (TJ) (Yanagawa *et al*, 1986). The cytoplasmic organelles are situated to one side of the cell and display low levels of secretory granules (Nagamine *et al*, 1990). These morphological characteristics indicate HSY cells resemble intercalated duct cells as opposed to acinar cells.

There are several features that make HSY an attractive model for engineering an artificial salivary gland as follows: (i) they exhibit TJs to maintain polarized monolayer organization (Yanagawa *et al*, 1986), which is critical for engineering a gland capable of fluid secretion (Aframian *et al*, 2002); (ii) they express amylase (Imai *et al*, 2004), indicating that they are able to maintain the phenotypical and functional characteristics necessary to engineer functional salivary gland tissue, which should secrete consistent levels of α-amylase *in vivo* (Joraku *et al*, 2005); (iii) they respond to muscarinic and β-adrenergic autonomic agonists to increase intracellular free calcium concentration ([Ca^2+^]_*i*_) and intracellular cyclic AMP ([cAMP]_i_), respectively (Patton *et al*, 1991), features that are essential for saliva secretion *in vivo* (Turner and Sugiya, 2002); and (iv) they can be easily transfected allowing for protein and genetic manipulation to modulate HSY cell growth and differentiation (Zhang *et al*, 2001). Previous studies demonstrated that transfection of the keratinocyte growth factor receptor gene to HSY cells induced differentiation and apoptosis, while suppressed tumor cell growth *in vitro* and tumor development *in vivo* (Zhang *et al*, 2001). It has been proposed that salivary intercalated ducts function as the reservoir for progenitor cells in the salivary gland (Nanduri *et al*, 2011). As HSY cells have similar morphological features to that of intercalated duct cells, it would be interesting to study whether non-transfected HSY cells may behave like progenitor stem cells when transplanted *in vivo*. To the best of our knowledge, no groups have used HSY cells for bioengineering studies. However, these cells are strong candidates for differentiation studies toward their use in an artificial salivary gland.

### HSG

HSG is a neoplastic intercalated duct cell line that was originated from an irradiated human submandibular gland (Sato *et al*, 1984). Histologically, HSG are cuboidal and conical in shape and have easily visible desmosomes with sporadic TJ complexes when grown on plastic (Shirasuna *et al*, 1981). Ultrastructurally, HSG have intercellular connections constituted of papillary infoldings, indicating secretory ability. Furthermore, they exhibit rough endoplasmic reticulum and Golgi complexes, indicating their ability to undergo exocytosis (Shirasuna *et al*, 1981).

HSG is a well-established *in vitro* model for salivary gland secretion, morphology, and regeneration (Kim *et al*, 2009; Wang *et al*, 2009). Several features indicate HSG as a potential source for developing an artificial salivary gland as follows: (i) they differentiate into acinar structures and express amylase when cultured on Matrigel (Royce *et al*, 1993; Hoffman *et al*, 1996; Vag *et al*, 2007); (ii) they have an innate capacity to increase [Ca^2+^]_*i*_ concentration in response to muscarinic and purinergic agonists (Nagy *et al*, 2007); (iii) they can be modulated by regulators of apotosis, providing researchers with a safety mechanism when working with neoplastic cells *in vivo* (Fukuda *et al*, 2007); and (iv) they are regulated by growth factor receptors (i.e., epidermal growth factor receptor (EGFR)) that may be used to promote repair or regeneration of salivary tissue (Ratchford *et al*, 2010).

Our group has found that treatment with Lipoxin A_4_ [a lipid mediator derived from arachidonic acid that is involved in the resolution of inflammation (Serhan *et al*, 1984)] inhibits immune cell binding to salivary epithelium, indicating that Lipoxin A_4_ serves as a “stop signal” for immune cell–mediated tissue damage (Chinthamani *et al*, 2011). These studies implicate HSG as an attractive model for co-culture studies (using different cell types) as they are able to bind to human T lymphocytes.

There are many drawbacks regarding the use of HSG when grown on plastic as follows: (i) they are unable to form TJs, making them incapable of attaining and maintaining the polarized monolayer organization required for fluid secretion and (ii) they do not express aquaporins (AQP, essential water channel proteins) AQP1 and AQP5, making them unfit for water transport studies (Delporte and Steinfeld, 2006). However, a recent study indicated that HSG grown on permeable supports coated with Matrigel are able to express TJ proteins (claudin-1, -2, -3, -4, occludin, JAM-A, and ZO-1) and AQP (AQP5) (Maria *et al*, 2011a, b). Nevertheless, future studies will be necessary to understand the barrier properties of this model, such as characterization of TJ morphology and in-depth analysis of monolayer permeability.

### SMIE and RSMT-A5

SMIE is an immortalized epithelial cell line derived from rat submandibular glands (He *et al*, 1990). This cell line was developed in 1990 and was originally named rat submandibular gland (RSMG); later, the cell line was re-named to SMIE because of the adenovirus (12S E1A gene product) used to immortalize the cells (He *et al*, 1998). SMIE cells were originally established to study polarized functions in salivary epithelium given their ability to form TJs when plated on collagen-coated permeable supports (He *et al*, 1990, 1998). Structurally, SMIE cells closely resemble salivary glandular epithelium with immature lumens; consequently, they appear relatively undifferentiated (He *et al*, 1998). SMIE cells express TJs (i.e., occludin and ZO-1) and adherens junctions proteins (i.e., E-cadherin), which are necessary for polarized functions in salivary epithelium (Michikawa *et al*, 2008). However, SMIE cells only express low levels of claudin-3 and lack other claudin family members (Michikawa *et al*, 2008). Claudin-3, -4, and -7 have been shown to play a role in salivary epithelium barrier function (Peppi and Ghabriel, 2004; Kawedia *et al*, 2007). SMIE cells display a *leaky* epithelium phenotype as a result of low protein levels of claudin-3 (He *et al*, 1998; Michikawa *et al*, 2008). This phenotype is exemplified with low transepithelial resistance (TER) values and high permeability to water-soluble, membrane-impermeant probes such as mannitol and dextran (He *et al*, 1998). However, SMIE cells are able to differentiate between various sized particles (4 kDa and 70 kDa dextran), allowing them to display selective barrier function and osmotically directed fluid transport (Michikawa *et al*, 2008). SMIE cells cultured together with IGF-1 increases TER (from approximately 3 to 40 Ωcm^2^) and permeability to 4 kDa fluorescein isothiocyanate dextran (FITC-dextran); these studies indicate that IGF-1 regulates paracellular barrier function in this cell line (Mitsui *et al*, 2010). SMIE cells have also been shown to secrete luciferase, a naturally non-secreted protein, when transfected with a pGL3-EGFSP construct (Aframian *et al*, 2007). These studies indicate that SMIE cells can be modulated to induce protein secretion. In summary, SMIE cells provide an intriguing model for investigating polarized functions of the salivary epithelium.

Another cell line of submandibular gland origin is the RSMT-A5 (A5), which was transformed into a cell line by treatment with 3-methylcholanthrene (Brown, 1973). This cell line displays a ductal epithelium phenotype and express a high density of α1-andrenergic receptors which show a metabolic behavior similar to smooth muscle cells (He *et al*, 1989). These results indicate that A5 cells could be used for receptor characterization and signaling studies. Previous studies indicate that A5 cells are difficult to transfect and therefore not suitable for protein secretion studies (Aframian *et al*, 2007). Together, these studies suggest that A5 cells could be used to study cell signaling in ductal epithelium.

## Immortalized cell lines

### SMG-C6 and SMG-C10

The SMG-C cell lines were isolated following transfection of a replication-defective simian virus (SV40) genome into rat submandibular acinar cells (Quissell *et al*, 1997). Only two clones, termed SMG-C6 and SMG-C10, were found to be both well differentiated and of epithelial origin (Quissell *et al*, 1997). Structurally, these cell lines are able to polarize because of their ability to form TJs and desmosomes (Quissell *et al*, 1997). Additionally, secretory features (i.e., domes, granules, and canaliculi) are observed in these cell lines (Quissell *et al*, 1997). Functionally, SMG-C6 respond to muscarinic and purinergic agonists (but not to α_1_ agonists) by increasing [Ca^2+^]_*i*_ (Liu *et al*, 2000). Furthermore, both SMG-C6 and SMG-C10 respond to β-adrenergic agonists by increasing [cAMP]_i_ (Liu *et al*, 2000). Of the two cell lines, SMG-C6 seems to be more cytodifferentiated than SMG-C10 owing to a greater quantity of secretory cellular structures and a more stable [Ca^2+^]_*i*_ release (Quissell *et al*, 1997). SMG-C6 cells are of acinar origin given the lack of cytokeratin 19 expression (which is expressed only in cells of ductal origin) (Castro *et al*, 2000). Both SMG-C6 and SMG-C10 lines are able to develop a high TER when grown on collagen-coated polycarbonate filters (Castro *et al*, 2000). We have not found studies documenting SMG-C6 and SMG-C10 growth on three-dimensional (3D) extracellular matrices. It would be interesting to determine whether they are able to fully differentiate under these conditions.

Both SMG-C6 and SMG-C10 serve as excellent models to study Na^+^ channels and expression of the Epithelial Na^+^ Channel protein (ENaC) given their ability to modulate Na^+^ transport in response to growth in culture medium lacking glucocorticoids or mineralocorticoids (Vasquez *et al*, 2009). Studies on the SMG-C10 cell line indicated that the cation channel transient potential vanilloid receptor 4 (TRPV4) was functionally connected to AQP5 volume (Aure *et al*, 2010). These studies indicate SMG-C10 as potential candidates for salivary cell volume regulation, which is an important feature to develop an artificial salivary gland.

A critical issue when using salivary cell lines is the fact that they are tumorogenic; therefore, it is important to control apoptosis in these cells lines. Previous studies using SMG-C6 demonstrated that apoptosis could be modulated (through a Fas-mediated pathway) (Aiba-Masago *et al*, 2001). Consequently, these cells could be used *in vivo* without the risk of uncontrolled cellular growth.

### Par-C10 and Par-C5

Following development of the SMG-C6 and SMG-C10 cell lines, another study was performed to isolate cells from a rat parotid gland (Quissell *et al*, 1998). Similarly to the SMG-C cell lines, parotid salivary cells were transfected with an origin-defective SV40 plasmid (Quissell *et al*, 1998). Both morphology and receptor-mediated calcium responses were used as a screening technique to monitor cell differentiation (Quissell *et al*, 1998). Both Par-C5 and Par-C10 cell lines form secretory granules, TJs, intermediate junctions, desmosomes, and microvilli (Quissell *et al*, 1998). When grown on plastic, Par-C10 form monolayers of cuboidal cells with thick extracellular matrices at their base while Par-C5 form layers of plump cells containing numerous intercellular lumen-like invaginations on their medial surfaces (Quissell *et al*, 1998).

Both Par-C5 and Par-C10 exhibit a significant elevation of [Ca^2+^]_*i*_ in response to cholinergic, muscarinic, and α_1_-adrenergic agonists (Liu *et al*, 2001). These cell lines increase [cAMP]_*i*_ in response to α_1_-adrenergic agonists similar to native tissues (Quissell *et al*, 1998). Additionally, they increase [Ca^2+^]_*i*_ in response to M_3_ muscarinic agonists (Bockman *et al*, 2001). No functional amylase expression has been observed in Par-C10 cells when grown on plastic or on growth-factor-reduced (GFR) Matrigel, although an interesting study on the Par-C three to nine clones reported a 16-fold increase in amylase content following incubation with rat serum (Zhu *et al*, 1998). However, further studies are needed to improve amylase production in these cell lines.

The Par-C10 cell line has been widely studied given its ability to develop the highest TER as compared to Par-C5, SMG-C6, and SMG-C10. This high TER makes them ideal candidates for studies in Ussing chambers (Turner *et al*, 1998). In fact, transepithelial anion secretion in Par-C10 cells has been well characterized and is regulated by basolateral α_1_-adrenergic and muscarinic cholinergic receptors and apical P2Y_2_ receptors (Turner *et al*, 1998). Furthermore, Par-C10 cells express Na^+^/H^+^ exchangers, Na^+^-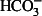 cotransporters, and anion exchange proteins on their basolateral surfaces (Demeter *et al*, 2009). These proteins, which regulate transepithelial transport, are sensitive to changes in both [Ca^2+^]_*i*_ and [cAMP]_i_ concentrations (Demeter *et al*, 2009). These studies demonstrate Par-C10 as an excellent model to characterize secretion, which is essential for the construction of an artificial salivary gland.

Our group has found that a 48-h treatment with the pro-inflammatory cytokines TNFα and IFNγ decrease TER in Par-C10 cells (Baker *et al*, 2008). This decrease is associated with increased permeability to normally impermeant molecules, disruption of TJ morphology, and a selective reduction of claudin-1 protein expression (Baker *et al*, 2008). TNFα and IFNγ are upregulated not only at the plasma level but also in salivary glands from patients with SS (Fox *et al*, 1994; Boumba *et al*, 1995). Understanding the mechanisms involved in cytokine-mediated disruption of TJs is important as it may lead to the development of a potential treatment for SS and to elucidate how TJs are modulated during saliva secretion. We recently demonstrated that RvD1 (a potent lipid mediator derived from docosahexaenoic acid (DHA), which promotes resolution of inflammation) blocked inflammatory responses caused by TNFα and enhanced epithelial integrity in Par-C10 monolayers (Odusanwo *et al*, 2012). These results suggest that RvD1 represents a novel therapeutic approach to block inflammatory responses in salivary glands. Additionally, they implicate RvD1 as an elicitor of acinar formation in salivary glands, which may lead to the improvement of culture conditions in salivary epithelial cells.

Par-C10 single cells form 3D acinar-like spheres when grown on GFR Matrigel. Under these conditions, Par-C10 acinar-like spheres are able to express TJs, AQP3, ion transporters, and M3 muscarinic receptors (Baker *et al*, 2010). Par-C10 cell spheres increased AQP5 expression when placed in a hypertonic medium (Baker *et al*, 2010). Furthermore, Par-C10 acinar-like spheres developed changes in potential difference in response to muscarinic agonist stimulation. These features make Par-C10 acinar-like spheres an intriguing model to characterize cell volume regulation and ion secretion in salivary epithelium.

## Primary cells

Currently, the use of primary cells represents the best option for creation of an artificial salivary gland because they more closely resemble native tissue (as compared to cell lines). Furthermore, they can be manipulated *in vitro* and then transplanted to the organism in which they were derived. However, there are critical problems associated with the ability of primary cells to achieve acinar formation *in vitro*. For instance, primary cells have the tendency to de-differentiate when grown on plastic (Szlavik *et al*, 2008). Additionally, they are apoptotic when dissociated into single cell suspensions (Walsh *et al*, 1998). Therefore, new approaches are necessary to maintain high viability of primary salivary cells in culture by modulation of apoptosis.

Previous studies indicated that single human parotid cells are able to differentiate into acinar and ductal structures when grown in a 3D environment (Joraku *et al*, 2007). Human parotid acinar structures expressed α-amylase, AQP5, and TJ proteins when grown on GFR Matrigel (Joraku *et al*, 2007). Another study indicated that these cells grown on hyaluronic acid hydrogels are able to assemble into acinar lobules, form TJs, develop central lumens, and express α-amylase (Pradhan *et al*, 2010). More recently, a group utilized a panel of cell surface markers (that are commonly used to isolate mesenchymal stem cells), for the localization of selective subtypes of salivary acinar cells (i.e., mucous and serous) (Maria *et al*, 2012). Collectively, these studies suggest that in the future we will be able to create polarized salivary structures capable of secretory function. Furthermore, we will be able to purify different salivary cell types to meet the needs of a given patient.

Prior work using Rhesus parotid gland cells grown on plastic demonstrated their ability to form apical TJs and basolateral expression of Na^+^/K^+^ pumps, indicating polarization. Also, these cells have the ability to be transfected with adenoviral vectors to express AQP1 (Tran *et al*, 2006), indicating the potential of this model for secretion studies. Another potential model for secretion studies involves the use of human SMG. These cells are able to develop TJs, microvilli, secretory granules, and a relatively high TER when grown on permeable supports (Tran *et al*, 2005). These results indicate that salivary cells are able to polarize, differentiate, and have the potential to be regulated by secretory agonists.

Few studies have shown that salivary cells can be transplanted into living organisms. For instance, human parotid cells grown on polyglycolic acid polymers were implanted subcutaneously into athymic mice (Joraku *et al*, 2005). Later, the polymer scaffolds were retrieved, and differentiated acinar structures were observed (Joraku *et al*, 2005). Another study transplanted labeled rat SMG into atrophic salivary glands (Sugito *et al*, 2004). After several weeks, the labeled SMG cells were detectable over a broad area of the atrophic gland and localized around the acini and ductal region (Sugito *et al*, 2004). The studies above demonstrate that salivary tissue could be transplanted and maintain differentiation. However, further studies are needed to show whether transplanted salivary cells are able to function in response to neurotransmitters.

## Progenitor cells

The use of stem and progenitor cells to create an artificial salivary gland is an exciting novel technique to improve current therapies. These cells have the potential to differentiate and replace damaged tissue, leading to the development of human stem cell therapy to restore the function of damaged salivary glands (Feng *et al*, 2009). However, there are no reliable methods to isolate sufficient numbers of human salivary gland stem cells for use in therapy (Lombaert *et al*, 2006).

Neonatal progenitor cells provide an attractive model for constructing an artificial salivary gland as they have been found to be involved in the regeneration of acinar, ductal, and myoepithelial salivary cells *in vitro* (Kishi *et al*, 2006). Isolating progenitor cells prior to radiation therapy, expanding the cells *in vitro*, and then transplanting the cells into the patient is an ideal therapeutic approach for treating patients whose salivary glands may potentially be damaged by irradiation (Lombaert *et al*, 2008). A transcription factor *Ascl3* (which is essential for the determination of cell fate, development, and differentiation of numerous tissues) and a structural protein K5^+^ (a cytokeratin that forms cytoskeleton intermediate filaments) have been established as markers of progenitor cells (Bullard *et al*, 2008; Knox *et al*, 2010). Transplanted *Ascl3*-expressing cells were able to induce differentiation and tissue repair in salivary glands, indicating that these cells are potent inducers of salivary gland regeneration (Knox *et al*, 2010). Furthermore, *Ascl3* was found to be expressed in ductal cells and demilune caps of submandibular glands. These studies indicate that they contribute to the maintenance of mature salivary tissue (Bullard *et al*, 2008). Conversely, K5 has been established as a marker for progenitor cells in tracheal and lung epithelial cells (Rock *et al*, 2009). Additionally, K5^+^ cells express Sox2, a transcription factor involved in the self-renewal of stem cells. However, K5^+^ cells make up a very small percentage (5–9%) of Sox2 cells (Lombaert *et al*, 2011). These studies suggest that K5 is not a well-established marker for salivary progenitor cells. Other studies demonstrated that cells located in the striated ducts of the salivary glands express other stem cell markers (i.e., CD24, CD49f, CD133, and c-Kit^+^) (Nanduri *et al*, 2011). Particularly, c-Kit was definitively established as a stem cell marker and therefore gained the highest priority, but flow cytometric analysis of cells obtained from submandibular glands indicated that only 0.058% of salivary cells expressed c-Kit (Nanduri *et al*, 2011). These results suggest that c-Kit is not an ideal marker for stem cell isolation in salivary glands, as it would not help to yield workable levels of stem cells. We believe that there are many types of progenitor cells in salivary glands that have not yet been characterized. Future studies are needed to identify more reliable progenitor cell populations and to investigate how they behave during tissue repair. These studies will be important for consolidation of the current methods to generate new functional salivary cells in culture.

α_6_β_1_ integrins have been found to be a marker for salivary progenitor cells in rats. Interestingly, this marker was only expressed after duct ligation (Okumura *et al*, 2003). These α_6_β_1_ integrins-expressing cells were used to establish an immortalized cell line of rat salivary progenitor cells (Yaniv *et al*, 2010). This cell line is able to differentiate into both acinar-like and ductal-like structures and has the ability to be modulated when grown on Matrigel-based 3D scaffolds; however, cells grown under these conditions display uncontrolled growth. Further studies are needed to determine whether the acinar-like and ductal-like structures generated from this cell line are capable of responding to salivary secretory agonists. Additionally, the uncontrolled cell growth has to be modulated before these structures can be used for transplantation *in vivo*.

The current techniques for isolating salivary stem cells continue to be the major limiting factor for their use in the creation of an artificial salivary gland. Further research is needed to develop a better marker for salivary stem cells. We are not aware of any studies that have been able to proliferate undifferentiated salivary stem cells *in vitro*. Once an isolating technique is developed, the next step will be growing and expanding the stem cells *in vitro*.

## Conclusions and perspectives

The development of an artificial salivary gland is sought after by many patients, but the ideal salivary cell (or perhaps a combination of cells) needs to be acquired before construction of a gland may be started. However, there are many factors regarding the cells that need to be considered. Cell lines exhibit unlimited growth and therefore present a problem when transplanted *in vivo*. The insertion of a suicide gene that responds to a certain agonist may be one possible way to modulate these tumorigenic cells for use *in vivo*. The ideal salivary cell would be isolated from autologous tissue in the form of primary or stem cells. Autologous salivary cells would be accepted by the host with no risk of rejection. However, these cells have not been grown efficiently *in vitro*. Future studies are needed to improve cell differentiation and secretory function. Additionally, it is important to understand whether these cells are able to survive in co-culture with other cell types that are relevant for salivary secretory function (i.e., myoepithelial cells). Hopefully, future research using these cell models will bring tangible benefits to those who suffer from hyposalivation.
